# Explaining Positional Differences of Performance Profiles for the Elite Female Basketball Players

**DOI:** 10.3389/fpsyg.2020.558750

**Published:** 2021-01-13

**Authors:** Zongpeng Zhai, Yongbo Guo, Shaoliang Zhang, Yuanchang Li, Hongyou Liu

**Affiliations:** ^1^School of Physical Education and Sports Science, South China Normal University, Guangzhou, China; ^2^Division of Sports Science and Physical Education, Tsinghua University, Beijing, China

**Keywords:** performance analysis, playing roles, game-related statistics, continental championships, generalized linear mixed model

## Abstract

The aim of the present study was to explore the differences in technical performances of players considering playing positions by controlling the effect of situational variables in each FIBA female continental basketball competition. Samples of 9,208 observations from 471 games in the America, Africa, Asia, and Europe Championships during 2013–2017 were collected and analyzed by generalized mixed linear modeling. The results showed that Centers from Europe had more 2-point made (ES = 0.69), 2-point attempted (ES = 0.79), and offensive (ES = 0.64) and defensive (ES = 0.48) rebounds than forward. Asian and European guards performed a fewer number of 2-point made (ES = 0.90; 0.91), 2-point attempted (ES = 1.06; 0.98), and offensive (ES = 1.30; 1.23) and defensive (ES = 0.93; 0.94) rebounds than Asian and European centers. African and Asian forward had more 2-point made (ES = 0.48; 0.50), 2-point attempted (ES = 0.50; 0.56) than guards. This study helps to better understand the technical demands of female basketball among different international competitions, which could pave a new way to analyze the development trend of female basketball and promoting specific training plans and game strategies for coaches and players.

## Introduction

Basketball is a complex team-based sport which needs the coordinated cooperation among teammates with different roles (Sampaio et al., 2006). With the development of basketball performance analysis, the research with teams as subject is increasingly unable to meet the needs of practical application. Therefore, many researchers have transferred their interest in the individual level (Sampaio et al., 2006, 2008; Delextrat and Cohen, 2009; Sindik and Jukić, 2011; Delextrat et al., 2015; Štrumbelj et al., 2015; Ferioli et al., 2018; Pion et al., 2018; Reina Román et al., 2019; Garcia et al., 2020). This information is useful not only for coaches and managers but also for players and researchers (Gòmez et al., 2009; Ibáñez et al., 2018). Specifically, it could allow coaches to set more rigorous and specific training schemes or technical–tactical game strategies according to the different playing positions at international competitions (e.g., Basketball World Cup or Olympic Game). Also, personnel scouts would benefit from this research by assessing athletes with different playing roles in order to optimize the recruitment process. From the point of athletes, research on playing roles is useful for their personal career with continuous and multiple developments concerning playing basketball in different manners. For the researchers, studies in relation to specific roles could bring the performance analysis of basketball into a microcosmic level (from teams to individuals), generating more specific practical value to practitioners.

Playing positions have been established based on specific function and characteristics on the court, and some studies have identified the differences between different playing positions from many aspects. In the view of anthropometrics, Ackland et al. (1997) and Erčulj and Bračič (2010) found that the guards had the least pronounced longitudinal dimensions, the forward were taller than guards while smaller than centers, and centers were the tallest group. Considering the physical and physiological demands, Delextrat and Cohen (2009); Scanlan et al. (2012), Ferioli et al. (2018), and Garcia et al. (2020) demonstrated that guards showed greater value than centers and forward in running ability like total distance covered or sprint or shuttle run test, revealing excellent ability in terms of aerobic and relative values of anaerobic power. These advantages with shorter recovery time allowed guards to execute high-intensity activities (HIA) frequently like repeated transition between offense and defense (Pojskić et al., 2015). However, Ferioli et al. (2020) found that the proportion of time spent conducting HIA for guards in the situation of ball possession was less than forward and centers, which means that guards undertook more tasks about regular dribbling (e.g., pushing the ball from backcourt to frontcourt) while forward and centers performed more offense with rapid movement and directly to the basket. Moreover, Ostojic et al. (2006) and Pojskić et al. (2015) reported centers and forward outperformed guards in absolute anaerobic power (e.g., vertical jump power), which implied that centers often utilize their somatic advantages to seize the space and execute some dirty works (e.g., box out for rebound or screen for pick and roll). Regarding the cognitive psychology, Sindik and Nazor (2011) compared the individual cognitive characteristics and perceived group cohesion considering basketball players in different playing positions. However, no significant difference was identified.

These differences in the above aspects have great impact on players’ performance, dominating their technical–tactical behaviors and reflected directly in the game-related statistics (box score) which is the key to setting up performance analysis of basketball. A limited number of studies documented the difference of game-related statistics considering the playing positions. Sampaio et al. (2006) used discriminant analysis to examine the differences in game-related statistics between basketball guards, forward, and centers playing in three professional leagues. The results showed the differences of technical performance in different leagues varied. For example, in the LCB league (Liga de Clubes de Basquetebol), defensive tasks like blocks and defensive rebounds were the main factors to discriminate guards from centers, while in the ACB (Asociacion de Clubs de Baloncesto) and the NBA (National Basketball Association), offensive tasks like assists and 3-point field goals became the key factors. Sindik and Jukić (2011) used the same approach to examine the differences of Croatian basketball players’ situational efficacy in relation to their playing positions and found that guards had higher efficiency in 3-point shots, while centers performed better in 2-point field goals. In addition, guards had more assists while centers dominated offensive and defensive rebounds. However, the sample volume of above studies was limited (12 games and 74 players, respectively), which means it was not very representative. Besides, the majority of current studies are still related to the development and assessment of men’s basketball which lead to the performance profiles of female basketball that lag far behind men’s basketball (Ibáñez et al., 2018; Zhai et al., 2020). More importantly, the performance indicators of individual are not stable when considering contextual variables such as gender and different continental competitions (Sampaio et al., 2004; Gómez et al., 2013; Ibáñez et al., 2018; Madarame, 2018b; Yi et al., 2018). Thus, further research is still needed to better understand the technical demands of female players in different playing positions.

Accordingly, the aim of this study was to identify the positional differences of technical performance within FIBA Female Continental Basketball Championships. The results of this study can be applied into developing more effective training programs or recruiting more suitable players in international female basketball competitions.

## Materials and Methods

### Sample

Continental basketball competitions are organized by the FIBA with the same rules. Specific information of teams in each continent is found in Table 1. Archival data of the 471 games in the Championships of female basketball of America, Africa, Asia, and Europe in 2013, 2015, and 2017 were obtained from the open-access official FIBA records (available at https://archive.fiba.com). Players who played less than ten minutes in a single game were excluded from the samples (Zhang et al., 2017), which lead to a sample of 9,208 game observations.

**TABLE 1:** The number of teams in FIBA Female Continental Basketball Championships from 2013 to 2017.

Continent	2013	2015	2017
Africa	12	12	12
Asia	12	12	8
America	10	10	10
Europe	16	20	16

### Validity and Reliability

In order to test the validity of data sets, a subsample of 20 games (final score differences equal to or less than 10 points) was randomly selected and observed by two experienced analysts (basketball coaches with more than 5 years of experience in basketball performance analysis). The results were compared with the gathered data on the website and perfect intra-class correlation coefficients (ICC = 1.0) were obtained for free-throws, two-point and three-point (both made and missed), offensive and defensive rebounds, turnovers, steals, blocked shots, and personal fouls. For assists and steals, the results were lower but still very acceptable (ICC = 0.83). There was a formal approval of all procedures from the Local Institution of Research Review Board.

### Procedure and Statistical Analysis

The game-related statistics were transformed to per-minute statistics (original statistics/min × 40 min) according to the amount of time players were on the court (Zhang et al., 2017). Continental players were divided into three groups according to playing positions in Table 2. Based on previous research (Sampaio et al., 2006), a total of 14 variables were selected to quantify the technical performance (Sampaio et al., 2006; Zhang et al., 2017). Definitions of these variables can be found in Table 3.

**TABLE 2:** Sample characteristics.

Continent	Centers	Forward	Guards	Total
	(*n* = 1,888)	(*n* = 3,599)	(*n* = 3,721)	(*n* = 9,208)
Africa	606	1,480	1,467	3,533
Asia	423	653	593	1,669
Europe	624	932	1,152	2,708
America	235	534	509	1,278

**TABLE 3:** Definition of selected technical game performance-related variables.

Variables	Definition
Two-point made (2ptM)	The number of two-point field goals that a player has made.
Two-point attempt (2ptA)	The number of two-point field goals that a player has attempted.
Three-point made (3ptM)	The number of three-point field goals that a player has made.
Three-point attempt (3ptA)	The number of three-point field goals that a player has attempted.
Free throws made (FTM)	The number of free throws that a player has made.
Free throws attempt (FTA)	The number of free throws that a player has attempted.
Offensive rebounds (OREB)	The number of rebounds that a player has collected, while they were on offense.
Defensive rebounds (DREB)	The number of rebounds that a player has collected, while they were on defense.
Total rebounds (TREB)	The total number of rebounds that a player has collected.
Assists (AST)	An assist occurs when a player completes a pass to a teammate that directly leads to a made field goal.
Personal fouls (PF)	The total number of fouls that a player has committed.
Turnovers (TOV)	A turnover occurs when the teams on offense loses the ball to the defense.
Steals (STL)	A steal occurs when a defensive player takes the ball from a player on offense, causing a turnover from offensive players.
Blocks (BLK)	A block occurs when an offensive player attempts a shot, and a defensive player tips the ball, blocking their chance to score.

Generalized mixed linear modeling was then realized with Proc Glimmix in the University Edition of Statistical Analysis System (version SAS Studio 3.6). The variables playing position, game outcome, game type, and team and opponent quality were included in the modeling as the fixed effects. Random effects for player name and team identity were added to account for repeated measurement on the players and teams. Separate Poisson regressions were run for each of the continental championships in the modeling, taking the value of each of the fourteen technical variables as dependent variables.

Playing position, game outcome, and game type were all included as nominal predictor variables in the modeling. Playing positions have three levels (Center, Forward, and Guard), game outcome with two levels (win and loss), and game type with two levels (balanced and unbalanced: point difference above and not above 10 points). The effect of team and opponent quality was estimated by the difference in the log of the team’s ranking in the Championships as a predictor (Yi et al., 2018).

Uncertainty in the true effects of the predictors was evaluated using non-clinical magnitude-based inferences as implemented in the spreadsheet accompanying the package of materials for generalized mixed modeling with SAS Studio (Hopkins, 2016). Estimated magnitudes and their confidence limits were expressed in standardized units and were assessed qualitatively with the following scale: <0.2 trivial, 0.2–0.6 small, 0.6–1.2 moderate, 1.2–2.0 large, >2.0 very large (Hopkins et al., 2009). Standardization was achieved by dividing the estimated effect by the between-player standard deviation, which was derived from the mixed model by adding the variance for the true differences between players to the team-to-team variance within players before taking the square root. Effects were deemed clear if the 90% confidence interval did not include substantial positive and negative values simultaneously. Clear effects were reported with a qualitative likelihood that the true effect was either substantial or trivial (whichever probability was greater) using the following scale: <0.5% most unlikely, 0.5–5% very unlikely, 5–25% unlikely, 25–75% possibly, 75–95% likely, 95–99.5% very likely, and >99.5% most likely (Hopkins et al., 2009).

## Results

**FIGURE 1:**
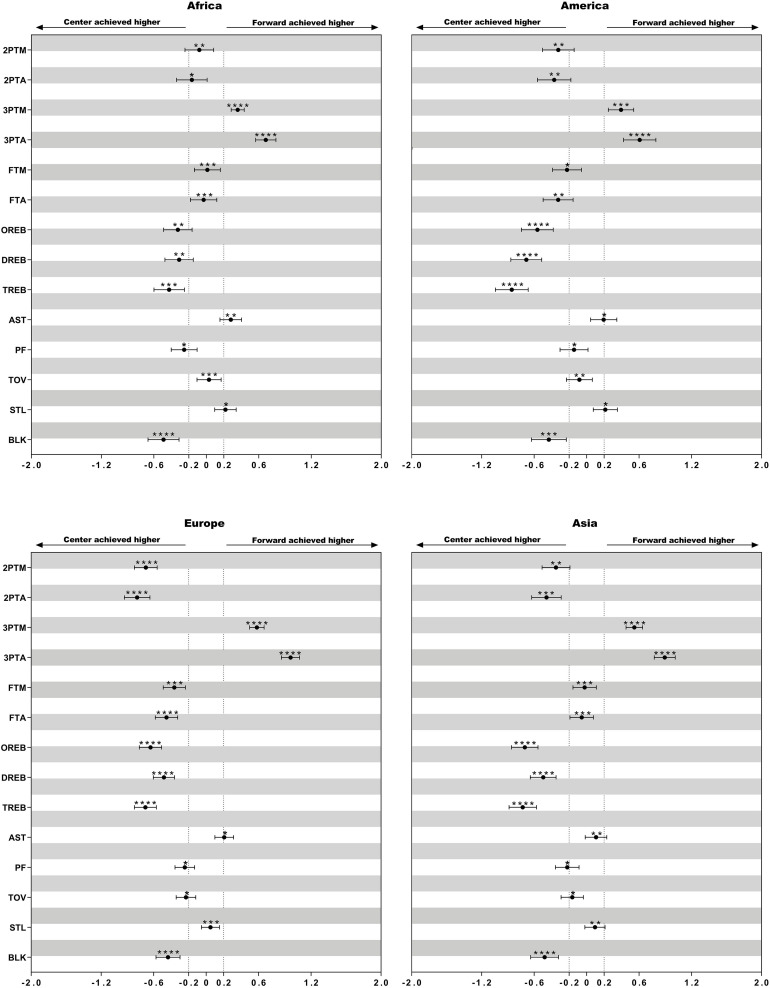
Standardized differences of technical game performance of center and forward. Bars are 90% confidence intervals. Asterisks indicate the likelihood for the magnitude of the true difference as follows: *possible; **likely; ***very likely; ****most likely. Asterisks located in the area between −0.2 and 0.2 denote for trivial differences.

**FIGURE 2:**
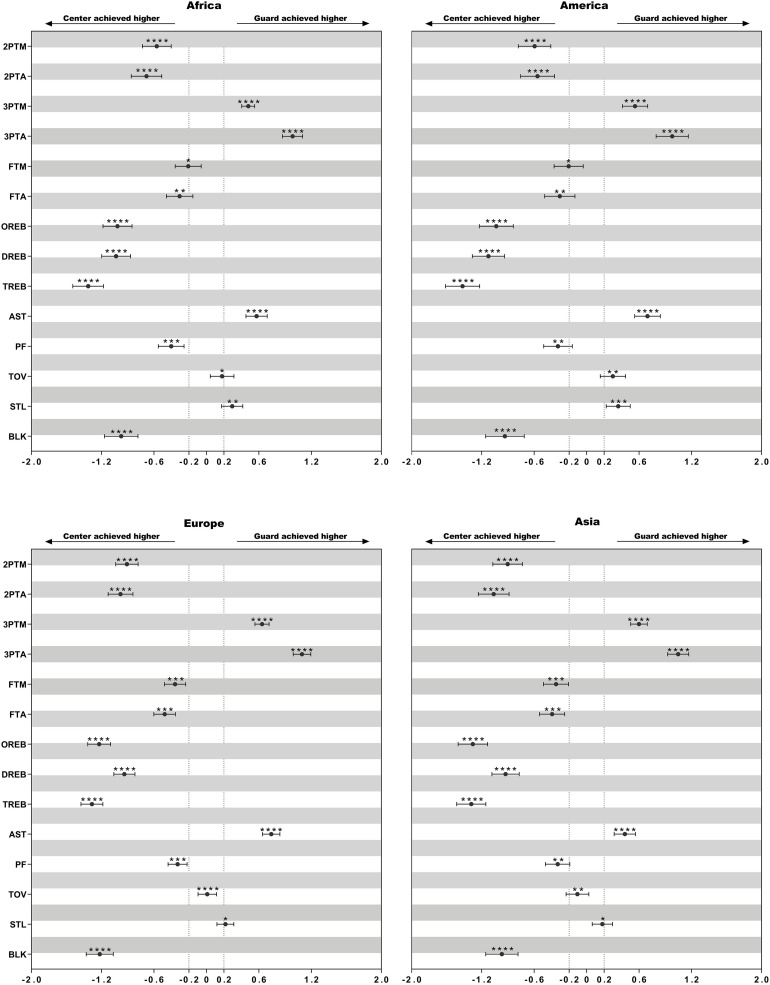
Standardized differences of technical game performance of center and guard. Bars are 90% confidence intervals. Asterisks indicate the likelihood for the magnitude of the true difference as follows: *possible; **likely; ***very likely; ****most likely. Asterisks located in the area between −0.2 and 0.2 denote for trivial differences.

**FIGURE 3:**
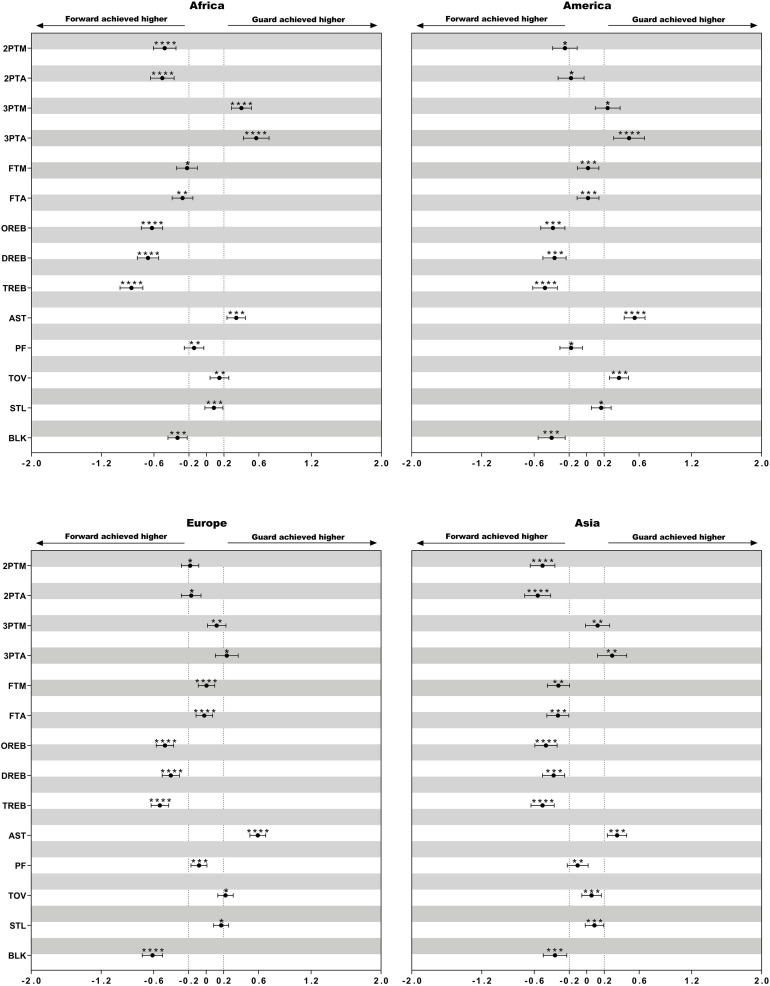
Standardized differences of technical game performance of forward and guard. Bars are 90% confidence intervals. Asterisks indicate the likelihood for the magnitude of the true difference as follows: *possible; **likely; ***very likely; ****most likely. Asterisks located in the area between −0.2 and 0.2 denote for trivial differences.

Descriptive statistics of technical variables of female basketball players of different playing positions in the listed four continental championships are presented in Table 4. The differences in the mean counts of performance-related statistics between playing positions within different continental championships are presented in Figures 1–3.

**TABLE 4:** Descriptive statistics of technical match performance of players from different playing positions of four continental championships.

Variable	Africa			Asia			America			Europe		
	G	F	C	G	F	C	G	F	C	G	F	C
2ptM	2.80 ± 2.86	4.46 ± 3.57	4.50 ± 3.49	2.79 ± 2.85	4.19 ± 3.58	5.52 ± 4.21	3.05 ± 2.72	3.84 ± 3.36	4.87 ± 3.82	2.98 ± 2.49	3.41 ± 2.93	5.49 ± 3.52
2ptA	7.33 ± 4.86	10.21 ± 5.8	10.65 ± 5.58	7.13 ± 4.94	9.90 ± 5.85	13.00 ± 7.50	7.94 ± 4.67	9.02 ± 5.50	10.92 ± 5.82	7.34 ± 4.19	8.10 ± 4.63	11.92 ± 5.41
3ptM	1.45 ± 2.12	0.70 ± 1.36	0.03 ± 0.30	1.50 ± 2.08	1.28 ± 2.03	0.19 ± 0.76	1.31 ± 1.80	1.03 ± 1.81	0.30 ± 0.96	1.31 ± 1.68	1.22 ± 1.73	0.19 ± 0.74
3ptA	5.13 ± 4.58	2.74 ± 3.30	0.39 ± 0.92	5.15 ± 4.48	4.38 ± 4.56	0.69 ± 1.60	4.71 ± 3.78	3.50 ± 4.13	1.14 ± 2.20	4.13 ± 3.12	3.78 ± 3.45	0.65 ± 1.50
FTM	2.26 ± 3.12	3.11 ± 3.57	2.85 ± 3.53	1.53 ± 2.49	2.33 ± 3.12	2.29 ± 2.96	2.44 ± 3.03	2.42 ± 3.22	2.93 ± 3.20	2.13 ± 2.89	2.08 ± 2.74	3.12 ± 3.44
FTA	3.53 ± 4.30	4.86 ± 4.98	4.73 ± 4.95	2.33 ± 3.54	3.42 ± 4.07	3.48 ± 3.85	3.41 ± 3.95	3.36 ± 4.11	4.38 ± 4.49	2.84 ± 3.59	2.89 ± 3.57	4.49 ± 4.45
OREB	1.39 ± 1.80	2.75 ± 2.90	3.75 ± 3.43	1.10 ± 1.80	1.92 ± 2.46	3.24 ± 3.08	1.39 ± 1.97	2.22 ± 2.63	3.69 ± 3.69	1.16 ± 1.60	1.95 ± 2.23	3.32 ± 2.84
DREB	3.18 ± 2.87	5.38 ± 3.88	6.86 ± 4.73	3.4 ± 2.95	4.54 ± 3.78	6.06 ± 4.18	3.93 ± 3.18	5.14 ± 3.71	7.42 ± 4.51	3.60 ± 2.83	4.82 ± 3.54	6.43 ± 3.76
TREB	4.57 ± 3.36	8.14 ± 5.18	10.6 ± 6.21	4.50 ± 3.70	6.46 ± 5.14	9.29 ± 5.67	5.32 ± 3.88	7.36 ± 4.92	11.12 ± 6.2	4.82 ± 3.82	6.74 ± 4.35	9.73 ± 4.86
AST	3.51 ± 3.45	2.47 ± 2.62	1.75 ± 2.09	3.75 ± 3.99	2.49 ± 3.57	2.20 ± 2.59	4.22 ± 3.54	2.42 ± 2.63	1.92 ± 2.34	4.15 ± 3.34	2.17 ± 2.25	1.79 ± 2.20
PF	3.63 ± 3.05	3.92 ± 2.95	4.89 ± 3.41	2.94 ± 2.76	3.26 ± 2.82	3.88 ± 3.26	3.37 ± 2.91	3.80 ± 2.94	4.39 ± 3.33	3.87 ± 2.83	4.14 ± 2.85	4.73 ± 3.25
TOV	4.34 ± 3.34	3.72 ± 2.92	3.75 ± 3.13	3.11 ± 2.89	3.07 ± 2.82	3.48 ± 2.83	3.95 ± 3.09	2.87 ± 2.58	3.11 ± 2.72	3.08 ± 2.49	2.58 ± 2.22	3.09 ± 2.47
STL	2.20 ± 2.41	2.04 ± 2.28	1.56 ± 2.02	2.03 ± 2.24	1.81 ± 2.36	1.61 ± 2.05	2.05 ± 2.24	1.71 ± 2.02	1.28 ± 1.78	1.65 ± 1.87	1.30 ± 1.65	1.25 ± 1.78
BLK	0.17 ± 0.62	0.43 ± 1.00	1.06 ± 1.90	0.21 ± 0.69	0.50 ± 1.11	0.98 ± 1.60	0.22 ± 0.74	0.54 ± 1.14	1.08 ± 1.62	0.16 ± 0.56	0.50 ± 1.03	1.07 ± 1.64

**G, guards; F, forward; C, centers; 2ptM, two-point made; 2ptA, two-point attempted; 3ptM, three-point made; 3ptA, three-point attempted; FTM, free throws made; FTA, free throws attempted; OREB, offensive rebounds; DREB, defensive rebounds; TREB, total rebounds; AST, assists; PF, personal fouls; TOV, turnovers; STL, steals; BLK, blocks.**

### Difference of the Performance Between Centers and Forward

Centers performed more 2ptA and 2ptM from America, Asia, and Europe than forward. All the regions’ forward had a higher number of 3ptA while this difference in Europe and Asia was more significant (ES: 0.89–0.97, moderately). Except for African centers, those from other three regions recorded a moderately higher number of TR (ES: 0.69–0.86).

### Difference of the Performance Between Centers and Guards

Asian and European centers achieved a moderately-to-large higher number of 2ptA, 2ptM, OR, and DR than that of guards (ES = 0.90–1.30). In all continental championships, centers showed a small-to-moderately fewer number of 3ptA, 3ptM, and AST (ES: 0.44–1.09).

### Difference of the Performance Between Forward and Guards

Forward from Africa and Asia showed higher values of 2ptA (ES = 0.53, small), 2ptM (ES = 0.49, small), OR (ES = 0.55, small), and DR (ES = 0.53, small) than guards. Guards from Africa and America had a greater number of 3ptM (ES = 0.32, small) compared with those in other two regions. In addition, American guards committed more turnovers than forward (ES = 0.37, small).

## Discussion

The aim of this study was to identify the positional differences of technical performance within FIBA Female Continental Basketball Championships by controlling game outcome, game status, teams, and opponent strength. Although the technical performance of players at different positions is similar across regions (e.g., centers always had more rebounds than that of guards, all the guards had more assists than that of centers), more detailed differences were identified. Therefore, this study may further explain positional differences in the FIBA international female continental basketball competitions.

### The Differences of Technical Performance Between Centers and Forward

European centers performed more 2ptM and 2ptA than forward, which was supported by Ibáñez et al. (2018) and Madarame (2018b). In order to enhance the offensive efficiency, European teams seemed to be more patient and tried to seek the best opportunity to shoot under a slow tempo. In addition, centers often execute more two-point shots because they stay near the basket and have the natural advantage of stature as well as a higher field-goal shooting percentage (Sampaio et al., 2006; George et al., 2009; Courel-Ibáñez et al., 2017). Therefore, coaches should be aware of the importance of 2-point shooting for European centers and set specific defensive strategies such as double team or front deny.

The study found that only African forward showed a trivial difference with centers in the number of 2ptM and 2ptA, which may reveal that they were played in a traditional style which emphasized on the 2-point field goals or middle-range shots. However, this type of traditional basketball did not bring any advantage to African teams because it has been corroborated by Madarame (2018b) that the number of 2ptM in African championships was the lowest among the four regions. In fact, those versatile players who can be efficiently qualified in multiple positions are more favored by coaches and managers because their malleability allows the recruitment processes and they line up more flexibly, especially in this era of “small ball” (Sampaio et al., 2006; Teramoto and Cross, 2010, 2018; Zhang et al., 2019a; Zhai et al., 2020). In this regard, personnel scouts should be cautious to add an African forward on the wish list.

To be self-evident, defensive rebound is the key part from defense to offense and it is also the necessary prerequisite to fast break or assists. In fact, defensive rebounds have become one of the vital indicators for winning in all levels of competitions and teams with a higher competitive level have a better ability to transform defensive rebounds to points (Gómez et al., 2006, 2008; Leicht et al., 2017; Paulauskas et al., 2018). Because of the advantage of playing position and activity area, centers always collect more defensive rebounds than forward (Sampaio et al., 2006; Zhang et al., 2019b). Our studies indicated that this difference was more significant in America, which may imply a whole team strategy that centers secured rebounds with fast break created by forward and guards. Therefore, opponents’ coaches could take advantage of these characteristics to mention forward to strive for offensive rebounds to get more scoring opportunities.

In addition, centers committed more fouls than forward. Generally, personal fouls were influenced by not only the player’s ability associated with individuals’ perception and judgment to the opponents’ action but also the playing positions (Sindik and Jukić, 2011). It seemed reasonable that centers committed more fouls than forward because they usually stay near the basket and are frequently challenged by opponents through various techniques and tactics. However, this trend could not be applied in American championships because no clear difference showed between these two positions. These findings are likely a consequence of established defensive strategy, which means American teams emphasized on the balance of defense in each position while others focused more on inside defense.

### The Differences of Technical Performance Between Centers and Guards

Guards from Europe and Asia secured less offensive rebounds than those of their centers. To some extent, offensive rebounds represented not only athletes’ motivation for competition but also a certain game strategy, which could generate more attacking opportunities (Ibáñez et al., 2018). In the defensive point of view, offensive rebounds represented the weakness of defensive players (Ibáñez et al., 2018). Thus, players should be encouraged to get more offensive rebounds when they play against European and Asian guards.

As a crucial indicator to game outcomes, assists were considered widely to be a measure of a player’s technique and a team’s overall tactical awareness (Gómez et al., 2008; Ibáñez et al., 2008, 2018; Paulauskas et al., 2018). In addition, assists were often performed by guards because they spent the most live playing time in possession of the ball (e.g., passing and ball handling) (Ferioli et al., 2020). The present study pointed out that the differences of assists between centers and guards in America and Europe were clearer than those in Asia and Africa, possibly because in more intense championships (e.g., America and Europe) (Madarame, 2018b), guards play the role of undertaking more responsibility for organizing and dominating the ball on offense (Sampaio et al., 2006). However, it is worth noting that there may be two different types of assists because American guards usually created opportunities by isolation, while European guards often relied on team tactics (Ibáñez et al., 2018). Thus, defensive deployment should be differentiated from one to the other when facing these two types of assists.

### The Differences of Technical Performance Between Forward and Guards

Teams or players with an excellent efficiency of 3-point goals could be a huge threat to the defender, forcing them into a dilemma between close-out to a 3-point line or drop for a dribble action (Sampaio et al., 2006; Teramoto and Cross, 2010, 2018; Zhang et al., 2019a). Generally, guards showed a better performance of 3-point shooting than forward (Sampaio et al., 2006; Sindik and Jukić, 2011). Our results indicated that compared with guards from Asia and Europe, those from America and Africa recorded a higher number of 3ptA and 3ptM than their forward. It may be attributed to the faster game rhythm where guards play the main role of controlling the game pace and dominating long-distance shooting (Ibáñez et al., 2018; Madarame, 2018b). Therefore, when playing against American or African teams, defensive players should place more emphasis on guards’ 3-point shots.

It has been proven that turnovers have an important effect on the game results, especially in women’s competitions (Leicht et al., 2017). As expected, guards had more turnovers than forward in all regions, which was supported by the study of Sampaio et al. (2006) and Vázquez-Guerrero et al. (2020) who stated that guards were more vulnerable to commit turnovers because they usually stay near the perimeter with higher pressure and performed the highest number of high-intensity accelerations and decelerations, especially in the game with a faster tempo (Ibáñez et al., 2018; Madarame, 2018a,b). Notably, American guards, compared with those in other three regions, recorded more turnovers than forward. This result is likely a consequence of the existing characteristics of game pace of American competitions and may also be attributed to the tactical strategies or technical habits that American guards spent more time in control of a live ball by holding or dribbling it (Madarame, 2018b; Ferioli et al., 2020). Consequently, American coaches should consider increasing the proportion of forward’ possession of the ball to relieve defensive pressure on guards. The main limitation of this study is that the positions of the players are determined through the team roster on the official website, and we hypothesize that players’ positions are fixed through the whole tournament. However, coaches may adjust the line-up to adapt to the demands of the game during playing time, which means that a guard could be arranged to play as a forward. Therefore, future studies are encouraged to identify players’ positions by more precise approaches such as video observation or other analysis software. Moreover, all the game-related statistics were obtained from the box score which only display regular indicators. Further studies may wish to excavate some data in relation to technical or tactical behavior such as shooting types, defensive habits, types of screens, and timing of cuts. Furthermore, all the comparisons were made specifically within each continental competition, which means further research can develop a comparison in a simultaneous tournament (e.g., Basketball World Cup and Olympic Games).

## Conclusion

In summary, the positional differences of technical performance in each four regions have been identified by this study. The greatest differences of offensive manner were between forward and centers from Europe. African centers and forward still insist on the traditional offense, which emphasizes 2-point field goals. Subsequently, in high-intensity competitions (America and Europe), guards executed more tasks of organization while those from America committed more turnovers. The results identified the differences of positional technique in female competitions, which may provide innovative perspectives on the pattern of modern female basketball games, as well as developing more specific training plans for coaches and players.

## Data Availability Statement

The original contributions presented in the study are included in the article/supplementary material, further inquiries can be directed to the corresponding author/s.

## Author Contributions

YG and HL contributed to the conception, design, and the examination of data of this research. SZ and YL contributed to the collection and examination of data. ZZ was responsible for writing. All authors contributed to the article and approved the submitted version.

## Conflict of Interest

The authors declare that the research was conducted in the absence of any commercial or financial relationships that could be construed as a potential conflict of interest.
